# Characterization of Nasal Potential Difference in *cftr* Knockout and F508del-CFTR Mice

**DOI:** 10.1371/journal.pone.0057317

**Published:** 2013-03-07

**Authors:** Emilie Lyne Saussereau, Delphine Roussel, Siradiou Diallo, Laurent Debarbieux, Aleksander Edelman, Isabelle Sermet-Gaudelus

**Affiliations:** 1 Institut Pasteur, Molecular Biology of the Gene in Extremophiles Unit, Department of Microbiology, Paris, France; 2 INSERM, U 845, Université Paris Descartes, Faculté de Médecine Necker Enfants-Malades, Paris, France; 3 Université Pierre et Marie Curie, Cellule Pasteur UPMC, Paris, France; University of Alabama-Birmingham, United States Of America

## Abstract

**Background:**

Treatments designed to correct cystic fibrosis transmembrane conductance regulator (CFTR) defects must first be evaluated in preclinical experiments in the mouse model of cystic fibrosis (CF). Mice nasal mucosa mimics the bioelectric defect seen in humans. The use of nasal potential difference (V_TE_) to assess ionic transport is a powerful test evaluating the restoration of CFTR function. Nasal V_TE_ in CF mice must be well characterized for correct interpretation.

**Methods:**

We performed V_TE_ measurements in large-scale studies of two mouse models of CF—B6;129 *cftr* knockout and FVB F508del-CFTR—and their respective wild-type (WT) littermates. We assessed the repeatability of the test for *cftr* knockout mice and defined cutoff points distinguishing between WT and F508del-CFTR mice.

**Results:**

We determined the typical V_TE_ values for CF and WT mice and demonstrated the existence of residual CFTR activity in F508del-CFTR mice. We characterized intra-animal variability in B6;129 mice and defined the cutoff points for F508del-CFTR chloride secretion rescue. Hyperpolarization of more than -2.15 mV after perfusion with a low-concentration Cl^-^ solution was considered to indicate a normal response.

**Conclusions:**

These data will make it possible to interpret changes in nasal V_TE_ in mouse models of CF, in future preclinical studies.

## Introduction

Cystic fibrosis (CF) is a lethal autosomal recessive disease that affects one in 2500 newborns in Caucasian population [1]. This disease is caused by mutations in the cystic fibrosis transmembrane conductance regulator (*CFTR)* gene, resulting in the production of a defective CFTR protein. CFTR is the main chloride (Cl^-^) channel in secretory epithelia and also acts as a regulator of sodium (Na^+^) transport, through inhibition of the ENaC Na^+^ channel [2]. Mutations in the *cftr* gene lead to the synthesis of a non-functional CFTR, causing dehydration of the airway surface liquid, thereby impeding mucociliary clearance and creating a favorable microenvironment for bacterial infections. The most frequent mutation results in deletion of the phenylalanine residue in position 508 (F508del-CFTR). This mutation leads to the retention of the F508del-CFTR protein in the endoplasmic reticulum and impaired function of any F508del-CFTR reaching the apical membrane [3]. There is currently no curative treatment for CF. Several strategies are currently being investigated for direct correction of the mutated CFTR defects, by rescuing trafficking defects [4]–[6] or rendering the mutated CFTR functional [5], [7]. However, these approaches must be tested in animal models. CF mice display nasal epithelium ionic transport abnormalities similar to those observed in humans with CF: abnormally high levels of Na^+^ absorption and an absence of Cl^-^ secretion in response to perfusion with a low-concentration Cl^-^ solution or a solution lacking this anion [8].

Transepithelial nasal potential difference (V_TE_) measurement is the most appropriate method for the *in vivo* exploration of ionic transport in CF [9]. This technique has been used in phase II clinical trials, as a means of assessing the restoration of CFTR function [10], [11]. It may also be very useful for preclinical studies assessing the efficacy of CFTR correctors or potentiators [6], [12]–[14].

However, V_TE_ measurement protocols differ between studies. Data have been obtained from pooled mice of different backgrounds [15], [16], for small numbers of mice and not for all V_TE_ parameters [15], [17]. Only two backgrounds are well characterized [14], [18]–[20]. Moreover, few data are available concerning variability within and between animals and no threshold for a significant, drug-related change has been validated. The aim of our study was i) to establish typical V_TE_ values, in the FVB and B6;129 backgrounds, for F508del-CFTR and *cftr^-/-^* mice, respectively, ii) to determine the repeatability of V_TE_ measurements, iii) to determine threshold V_TE_ values distinguishing between the CF and WT electrophysiological responses in F508del-CFTR mice. These data should improve the use of CF mice in preclinical studies.

## Materials and Methods

### Mouse models

We studied male and female B6;129-CFTR^tm1-Unc^ (*cftr* ^-/-^) mice, FVB mice homozygous for the F508del-CFTR mutation (F508del-CFTR) and their respective wild-type (WT) control littermates. Mice were obtained from CDTA (Orléans, France) and housed at the SPF Animal Care Facility of Necker University. The mice were 8 to 16 weeks old and weighed 20 to 28 g. All mice were fed a fiber-free diet. Colopeg (17.14 g/l; Bayer Santé Familiale, France) was administered to CF mice to prevent intestinal obstruction. Animal protocols were approved by the local ethics committee dealing with animal welfare and conformed to European Community regulations for the use of animals in research (authorization no. P2.AE.092.09).

### V_TE_ measurements

The method for nasal potential difference measurement was adapted and miniaturized from that developed by our group for use in young children [21]. Mice were anesthetized by an intraperitoneal injection of ketamine (133 mg/kg; IMALGENE 1000, MERIAL, France) and xylazine (13.3 mg/kg; Rompun 2%, BayerPharma, France). Mice were positioned on a 45° tilt board and a paper pad was placed under the nose to prevent the mice suffocating.

Transepithelial potential was measured between an Ag/AgCl reference electrode and an Ag/AgCl exploring electrode. The two Ag/AgCl electrodes were connected to a high-impedance voltmeter (LOGAN Research Ltd, United Kingdom). The reference electrode was connected to a subcutaneous needle with an agar bridge. The exploring Ag/AgCl electrode was connected to the nasal mucosa through a double-lumen polyethylene catheter (0.5 mm in diameter) inserted into the right nostril to a depth of 4 mm. Recordings were made every second, during continuous flow, at a rate of 0.15 ml/h, of the initial Cl^-^ solution (140 mM NaCl, 6 mM KCl, 10 mM HEPES, 10 mM glucose, 1 mM MgCl_2_, 2 mM CaCl_2,_ pH adjusted to 7.4 with NaOH) through the lumen directly connected to the exploring electrode. The following solutions were perfused through the second lumen at a flow rate of 1.5 ml/h: (1) Cl^-^ solution for basal measurement, (2) 100 µM amiloride in Cl^-^ solution (Sigma-Aldrich, USA), to block ENaC Na^+^ absorption (3) a low-Cl^-^ solution (140 mM sodium gluconate, 6 mM potassium gluconate, 10 mM HEPES, 10 mM glucose, 1 mM MgCl_2_, 6 mM calcium gluconate_,_ pH adjusted to 7.4 with NaOH) containing amiloride (100 µM) to drive Cl^-^ secretion. Each solution was perfused for at least three minutes. Stability for at least one minute was required before each change in perfusion. The values analyzed were the means of the last 30 seconds.

Three parameters were investigated during transepithelial nasal potential difference (V_TE_) measurements: (1) the stable maximal baseline V_TE_, which was obtained after the equilibration of transepithelial ion transport with Cl^-^ solution, and the successive net voltage changes between (2) baseline V_TE_ and the Cl^-^ solution containing 100 µM amiloride (ΔV_TE Amil_); (3) Cl^-^ solution with amiloride and low-Cl^-^ solution with amiloride (ΔV_TE LowCl_^-^).

### Inhibitors and activators

CFTR is a cAMP-dependent channel. We therefore used forskolin (Sigma-Aldrich, USA), an adenylate cyclase activator, in B6;129 and FVB WT mice, for the specific activation of CFTR. The V_TE_-based outcomes were net voltage differences between 100 µM amiloride in low-Cl^-^ solution and 100 µM amiloride plus 10 µM forskolin in low-Cl^-^ solution (ΔV_TE Forsk._).

We used various inhibitors to identify the channels participating in the low-Cl^-^ response in B6;129 WT mice: the CFTR-specific inhibitor thiazolidone (Inh-172) (Calbiochem, Germany), 5 µM; niflumic acid, a calcium-dependent Cl^-^ channel inhibitor (Sigma-Aldrich, USA), 100 µM and zinc chloride, a voltage-dependent Cl^-^ channel inhibitor, (Fluka, USA), 50 µM. These inhibitors were tested in low-Cl^-^ solution containing amiloride (100 µM), and the V_TE_-based outcomes recorded were net voltage changes after perfusion of the given inhibitor in low-Cl^-^ solution (ΔV_TE Inh._).

We investigated the involvement of channels other than CFTR in Cl^-^ secretion, using broad-spectrum inhibitors of anion transporters: diphenylamine-2-carboxylic acid (DPC) and disodium 4,4′-diisothiocyanatostilbene-2,2′-disulfonate (DIDS) were tested on six B6;129 WT mice, in low-Cl^-^ solution containing amiloride (100 µM) and Inh-172 (5 µM). The V_TE_-based outcomes measured were net voltage changes between amiloride plus 5 µM Inh-172 in low-Cl^-^ solution and amiloride plus 5 µM Inh-172 plus 200 µM DIDS (Fluka, USA) or 200 µM DPC (Fluka, USA) in low-Cl^-^ solution.

We assessed the effects of solvents (DMSO, ethanol, NaOH and KHCO_3_) on voltage. None of the solvents altered Cl^-^ secretion at the concentration used to dissolve the various inhibitors (data not shown).

### Statistical analysis

The values obtained were not normally distributed. The electrophysiological norms for ΔV_TE_ parameters are therefore expressed as medians with interquartile ranges (IQR).

We compared the V_TE_ values obtained between groups in Mann-Whitney tests.

We assessed the effects of inhibitors and activators in Wilcoxon paired signed-rank tests comparing V_TE_ data recorded after the perfusion of low-Cl^-^ solution and V_TE_ data recorded after the perfusion of low-Cl^-^ solution plus inhibitor/activator, except for DIDS effects for which V_TEInh-172_ and V_TEDIDS_ data were compared.

The difference between two measurements for a single mice group was evaluated with the non parametric Wilcoxon paired signed-rank test. Repeatability was evaluated by the Bland-Altman method [22]. For each mouse, the difference between two measurements was calculated and plotted against the mean of the two measurements. We determined whether the differences were normally distributed in Kolmogorov-Smirnov and Shapiro-Wilk normality tests. The bias was estimated by calculating the mean of all differences between the two measurements. If this mean is not close to zero, the two assays are considered to give different results. The limits of agreement were defined as the bias ± 1.96 SD.

A case-control analysis was carried out to optimize the discrimination between control mice and F508del-CFTR mice. Cutoff points were determined from the receiver operating characteristics (ROC) curve. For each parameter, we ranked the values for WT and F508del-CFTR mice. The percentage of the WT mice effectively included in each rank indicates the sensitivity of the test; the percentage of F508del-CFTR mice effectively not included in each rank indicates the specificity of the test. Cutoff points were defined as the rank associated with the best positive likelihood ratio of sensitivity/(1-specificity), favoring specificity.

## Results

### Protocol implementation

Perfusion with Cl^-^ solution induced depolarization in WT mice, by about 8.7 mV (IQR 4.4) in B6;129 mice (*n*  =  35) and 10.3 mV (IQR 4.1) in FVB mice (*n*  =  12) (data not shown). We therefore recorded baseline V_TE_ after perfusion with Cl^-^ solution.

Forskolin induced no significant increase in Cl^-^ secretion in either B6;129 (*n*  =  10) or FVB (*n*  =  9) WT mice (Table S1). Furthermore response to forskolin perfusion did not discriminate between WT and CF mice (Table S1). We therefore decided not to test forskolin after perfusion with a low-Cl^-^ solution.

Neither niflumic acid (*n*  =  6) nor zinc ions (*n*  =  6) significantly inhibited chloride conductance. Inh-172 decreased Cl^-^ secretion significantly, by 2.3 mV (*n*  =  6; *p*  =  0.03). Both DIDS and DPC induced a significant additional depolarization, of about 2.2 mV (*n*  =  6; *p*  =  0.03, for both). As CFTR is sensitive to the broad-spectrum inhibitor DPC [23], [24], but not to DIDS [24], [25], we decided to inhibit Cl^-^ secretion by the following sequence: (1) Inh-172 in low-Cl^-^ solution, to inhibit CFTR specifically, (2) DIDS in low-Cl^-^ solution containing Inh-172, to inhibit potential anion transporters other than CFTR.

### Nasal potential difference values

#### Typical values in B6;129 WT and CF mice

**Figure 1:**
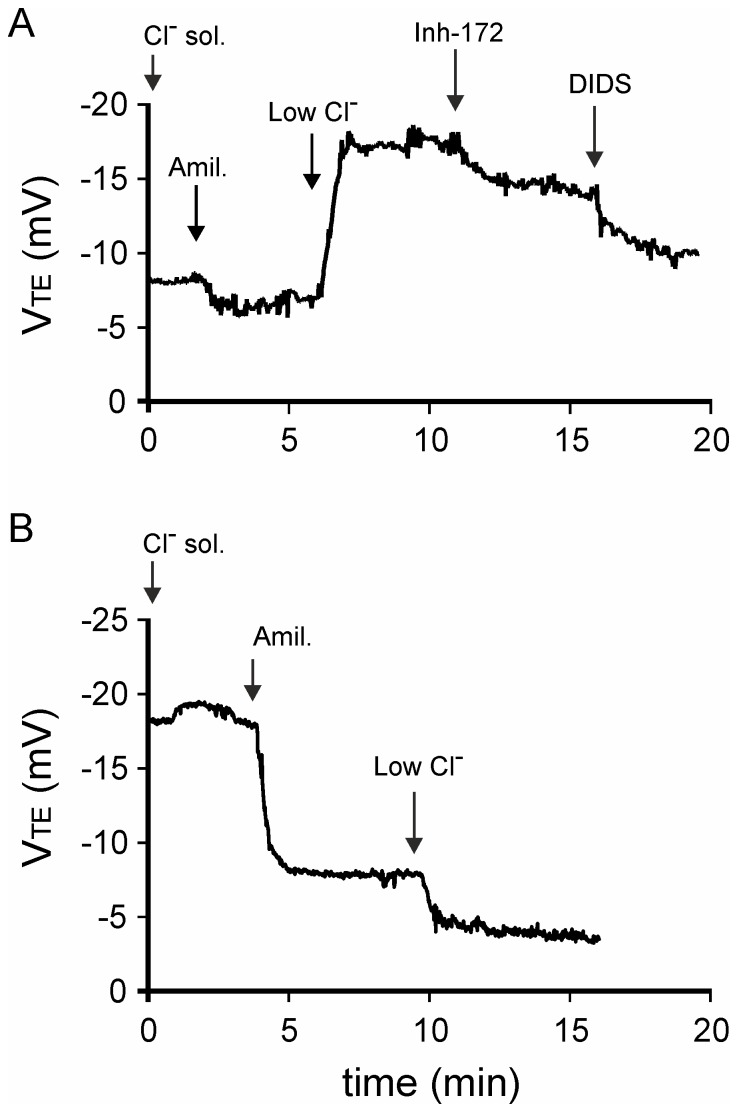
Representative V_TE_ recordings. The recordings obtained with the final protocol are shown for WT (A) and *cftr* ^-/-^ B6;129 mice (B). Three phases was observed: baseline V_TE_ after Cl^-^ solution perfusion, V_TEAmil._ after the addition of amiloride (Amil.) and V_TELowCl_^-^ after the replacement of Cl^-^ solution with a solution of low Cl^-^ concentration (LowCl^-^). The inhibitory effect on Cl^-^ secretion of inhibitor-172 and inhibitor-172 plus DIDS was demonstrated in WT mice.

Transepithelial nasal potential difference (V_TE_) measurements were performed in 50 WT and 50 *cftr* knockout mice (*cftr* ^-/-^); representative recordings are shown in Figure 1. Sex had no effect on any of the V_TE_ parameters in either of these groups. The *cftr* ^-/-^ mice had higher levels of sodium transport than WT mice, as shown by the higher baseline V_TE_ and much more pronounced response to amiloride perfusion, and an absence of chloride transport, as shown by the lack of response to perfusion with low-Cl^-^ solution (Table 1). WT mice displayed strong hyperpolarization during perfusion with the low-Cl^-^ solution (-7.8 mV (IQR  =  3.8 mV)), which was inhibited by 20% (*p*<0.0001) with Inh-172 and an additional 26% (*p*<0.0001) with DIDS. The *cftr* ^-/-^ mice did not respond to Inh-172, but displayed additional depolarization, by 3.1 mV (IQR  =  3.1 mV, *p*<0.0001) after DIDS perfusion.

**Table 1:** Typical values for B6;129 and FVB V_TE_ mice.

	Typical values				Comparison (*p*-value)			
	B6;129		FVB		B6;129	FVB	WT	CF
	WT	cftr^-/-^	WT	F508del-CFTR	(WT / cftr^-/-^)	(WT / F508del-CFTR)	(B6;129 / FVB)	(cftr^-/-^ / F508del-CFTR)
Baseline VTE (mV) Median (IQR)	–4.9 (3.5) *n* = 50	–20.9 (6.5) *n* = 50	–4.2 (5.2) *n* = 25	–13.3 (5.4) *n* = 50	<0.0001	<0.0001	ns	<0.0001
ΔVTE Amil (mV) Median (IQR)	1.6 (1.3) *n* = 50	9,1 (4.2) *n* = 50	1.5 (1.7) *n* = 25	7.1 (4.2) *n* = 50	<0.0001	<0.0001	ns	0.002
ΔVTE Low Cl- (mV) Median (IQR)	–7.8 (3.8) *n* = 50	3.0 (4.1) *n* = 50	–4.7 (5.3) *n* = 25	0.8 (2.4) *n* = 50	<0.0001	<0.0001	0.003	0.001
ΔVTE Inh-172 (mV) Median (IQR)	1.6 (2.3) *n* = 50	0.9 (1.7) *n* = 50	1.6 (3.1) *n* = 17	0.2 (2.0) *n* = 45	0.02	0.05	ns	ns
ΔVTE DIDS (mV) Median (IQR)	2.0 (1.7) *n* = 38	3.1 (3.1) *n* = 32	2.4 (2.8) n = 9	2.9 (3.9) *n* = 6	0.003	ns	ns	ns

Values are given as the medians ± interquartile range (IQR) for WT and *cftr^-/-^* B6;129 mice and WT and F508del-CFTR FVB mice. Inhibitory effects were assessed for Inh-172 and for DIDS. Mann-Whitney tests were used to compare groups.

#### Nasal potential difference in FVB and B6;129 mice

Transepithelial nasal potential difference (V_TE_) measurements were performed on 50 F508del-CFTR and 25 WT FVB mice (Table 1). No V_TE_ difference was observed between males and females. Like *cftr* ^-/-^ mice, F508del-CFTR mice had higher levels of sodium transport and absent or lower levels of chloride secretion than their WT littermates.

WT B6;129 and FVB mice had similar levels of Na^+^ transport, as shown by their similar baseline V_TE_ and ΔV_TE Amil_ values. By contrast, WT FVB mice had significantly lower levels of Cl^-^ secretion, as shown by their ΔV_TE Low Cl_^-^ values, which were lower than those of B6;129 mice by a factor of about 1.5 (*p*  =  0.0025). The contribution of the CFTR was similar in mice of both backgrounds, because Inh-172 treatment resulted in significant inhibition of 1.6 mV (IQR 3.1, *p*  =  0.0039) in FVB mice and 1.6 mV (IQR 2.3) in B6;129 mice. In WT FVB mice, DIDS treatment resulted in an additional inhibition, by 2.4 mV (IQR 2.8, *p*  =  0.0078), corresponding to 51% inhibition of chloride secretion.

F508del-CFTR mice had lower levels of sodium transport than *cftr* ^-/-^ mice, as shown by their significantly lower baseline V_TE_ and ΔV_TE Amil_ values (*p* < 0.0001 and *p*  =  0.002). As in *cftr* ^-/-^ mice, no chloride secretion was observed.

### Intermeasurement repeatability

**Figure 2:**
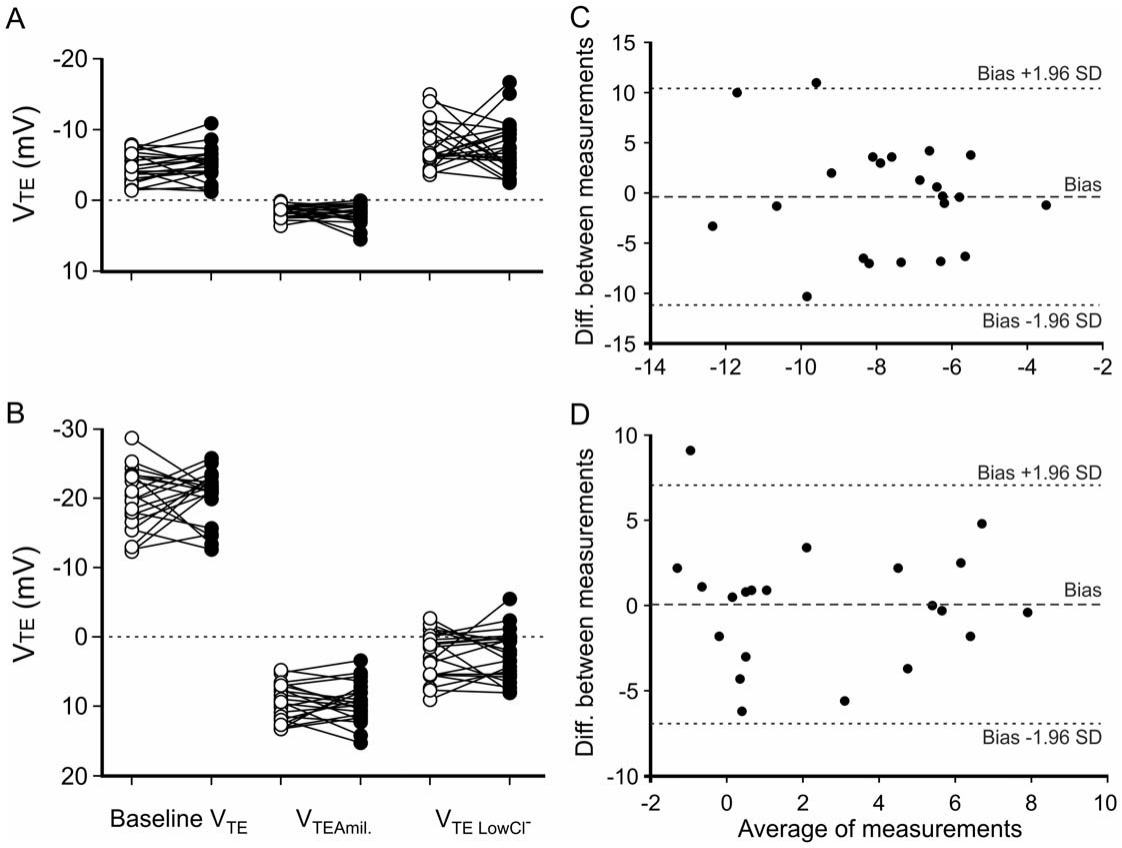
Reproducibility between two measurements in B6;129 mice. The first measurement (○) was obtained at least 7 days before the second measurement (•) on 22 WT (A) and 21 *cftr* ^-/-^ mice (B). The difference between the two ΔV_TELowCl_^-^ values was plotted against their mean, as described by Bland and Altman, for the 22 WT (C) and 21 *cftr* ^-/-^ (D) mice.

Figure 2 shows the results of two measurements taken one to four weeks apart in 22 WT mice (A) and 21 *cftr* ^-/-^ mice (B).

There was no significant difference between the two series of measurements for baseline V_TE_, ΔV_TE Amil_ and ΔV_TE Low cl_^-^, in either WT or *cftr* ^-/-^ mice, as assessed by Wilcoxon paired signed-rank tests.

The repeatability of the test was assessed by the Bland-Altman method for baseline V_TE_, ΔV_TE Amil_ and ΔV_TE Low cl_^-^, in both WT (Figure 2C) and *cftr* ^-/-^ mice (Figure 2D). Differences between the two measurements were normally distributed and did not vary as a function of their arithmetic values. Intra-animal variability was defined by the limits of agreement, both in WT and *cftr* ^-/-^ mice, and for all V_TE_ parameters (Table 2). Bias — i.e. the mean differences — were close to zero in both WT and *cftr* ^-/-^ mice (Table 2).

**Table 2:** Parameters of the Bland-Altman plot.

	Bias	SD	Limits of agreement (Bias + 1.96 SD/Bias - 1.96 SD)	% outside the limits
WT				
Baseline VTE	0.27	2.12	4.4 / –3.9	4.5
Δ VTE Amil	–0.35	1.57	2.7 / –3.4	4.5
ΔVTE Low Cl-	–0.37	5.51	10.4 / –11.2	4.5
*cftr^-/-^*				
Baseline VTE	0.51	5.53	11.3 / –10.3	0
ΔVTE Amil	–0.16	3.05	5.8 / –6.1	4.8
Δ VTE Low Cl-	0.06	3.57	7.1 / –6.9	4.8

Values were determined for baseline potential difference (V_TE_), amiloride response (ΔV_TEAmil._) and low Cl^-^ solution response (ΔV_TELowCl_^-^), from the Bland and Altman plot, for two measurements on the same 22 WT and 21 *cftr* ^-/-^ mice.

### Determination of the cutoff points separating FVB WT and F508del-CFTR mice, for V_TE_ parameters

**Figure 3:**
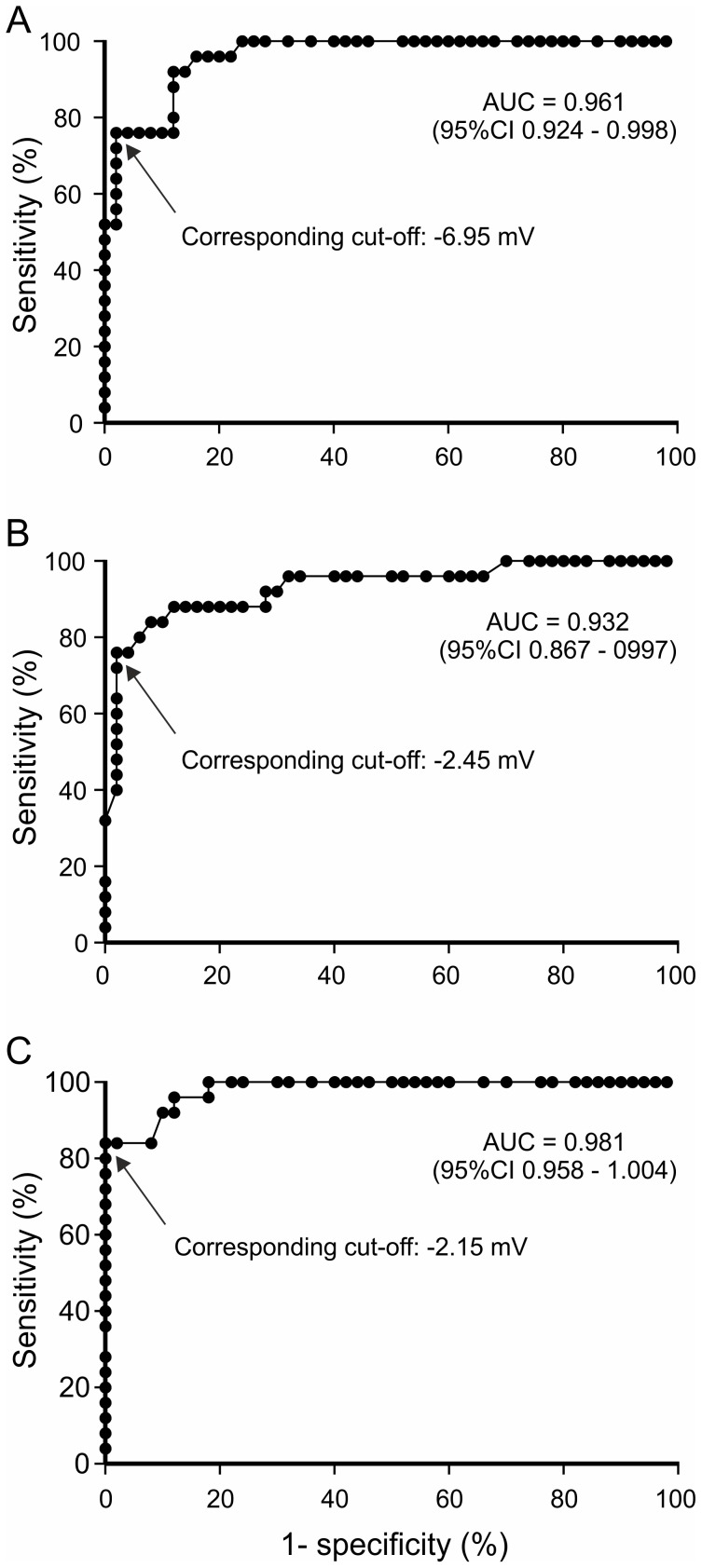
Cutoff point determination for nasal potential difference parameters distinguishing between FVB WT and F508del-CFTR mice. Receiver operating characteristics (ROC) curves for (A) baseline potential difference (V_TE_), (B) amiloride response (ΔV_TEAmil._) and (C) low Cl^-^ solution response (ΔV_TElowCl_^-^), for WT and F508del-CFTR FVB mice are shown. AUC: area under the curve; 95% CI: 95% confidence interval. Cutoff points were determined by the best positive likelihood ratio of sensitivity/(1-specificity).

Figure 3 shows the receiver operating characteristics (ROC) curve obtained with V_TE_ data for 25 FVB WT and 50 F508del-CFTR mice. All the areas under the curve (AUC) were very close to 1.00, demonstrating a high level of discrimination between WT and F508del-CFTR mice. This case-control analysis led to the definition of the following cutoff points, indicative of normal ion transport: baseline V_TE_ value > -6.95 mV; ΔV_TE Amil._ value < 2.45 mV and ΔV_TE LowCl_^-^ value < -2.15 mV.

## Discussion

In this study, we established values for nasal potential difference endpoints in mice. We studied baseline V_TE_, response to amiloride and to low-Cl^-^ solution in 129;B6 *cftr* ^-/-^ and FVB F508del-CFTR mice, and their respective controls. In both CF models, ENaC activity was much higher than in the corresponding WT (Baseline V_TE_: ∼ 3.5 times higher and ΔV_TEAmil_: ∼ 5 times higher) and Cl^-^ secretion was abolished. We assessed the variability of this test on Bland-Altman plots and determined the first cutoff points for distinguishing between WT and CF mice. These cutoff points are important for the evaluation of CFTR transport restoration in preclinical studies evaluating CFTR correctors or potentiators [6], [12]–[14].

### Protocol implementation

Protocols for V_TE_ determinations in mice vary considerably, with differences in perfusion flow rate of perfusion (from 0.018 ml/h to 3 ml/h), the position of the catheter in the nostril (2 mm to 5 mm into the nostril) and the nature of the solution used (Ringer’s solution, Krebs solution, other phosphate or HEPES solutions) [14]–[20], [26], [27]. The sequences of the solutions used also differ markedly between protocols: i) Some groups initially perfuse with the Cl^-^ solution until stabilization is achieved, before recording baseline V_TE_ [19], [20], whereas other groups do not carry out this perfusion step [17], [18]. ii) The use of a CFTR activator (forskolin, isoproterenol) and CFTR-specific inhibitors during perfusion with the low-Cl^-^ solution is not systematic [14], [17], [18]. We defined a protocol taking into account the following points: i) WT mice displayed depolarization after initial Cl^-^ solution perfusion. We therefore decided to perfuse the epithelium with this solution until stabilization was achieved, before recording baseline V_TE_. ii) As no additional hyperpolarization was observed after the perfusion of forskolin solution, in either of the WT backgrounds, we decided not to use forskolin. Similar results were reported by Brady *et al.* for mice of the BALB/cJ and C3H/HeJ backgrounds [15]. Moreover, the response to forskolin (which increase cAMP level), had been shown to be small in WT mice and cannot reliably used to distinguish between WT and CF genotypes because CF mice display a small response similar to that of WT mice [15], [16], [18], [28].

We further characterized Cl^-^ secretion with various inhibitors. Inh-172, a specific inhibitor of CFTR, and DIDS, a broad-spectrum inhibitor, had significant effects. Neither inhibitors of Ca^2+^- (niflumic acid) nor inhibitors of voltage-dependent Cl^-^ channels (zinc chloride) affected Cl^-^ conductance. This suggests that neither Ca^2+^-dependant Cl^-^ channels (CaCC) nor voltage-gated channels were active in the murine nasal epithelium in basal conditions.

### VTE values in mice of the two backgrounds

The lack of well characterized backgrounds for V_TE_ measurements led us to investigate the FVB and B6;129 backgrounds in detail. Baseline V_TE_ was significantly higher in CF than in WT mice, consistent with the differences observed between CF patients and healthy people [21], [29]. The response to amiloride perfusion was also about five times stronger in the CF models, *cftr* ^-/-^ and F508del-CFTR mice, than in WT mice. However, this difference was smaller in F508del-CFTR mice than in *cftr* ^-/-^ mice, suggesting that the F508del-CFTR protein may have retained some of its ENaC channel-regulating activity. The corresponding WT had similar baseline V_TE_ and ΔV_TEAmil_ values. Thus, genetic background is not responsible for effects on sodium transport.

In terms of Cl^-^ secretion, mice of the two backgrounds were similarly sensitive to Inh-172 and DIDS. However, an interesting difference between these two backgrounds was that chloride secretion was almost entirely inhibited by Inh-172 plus DIDS in FVB mice, whereas secretion was inhibited by less than 50% in B6;129 mice (residual V_TE_  =  0.7 mV vs. 4.2 mV, respectively). Thus, in addition to CFTR, there is a DIDS-insensitive Cl^-^ pathway in mouse nasal epithelia, but the contribution of this pathway differs considerably between genetic backgrounds.

Hyperpolarization by at least -1.9 mV was observed in 10 % of FVB F508del-CFTR mice, but no B6;129 *cftr* ^-/-^ mice, in response to perfusion with a low-chloride solution. This demonstrates that CFTR activity is therefore responsible for chloride secretion in wild-type B6;129 mice and also that F508del-CFTR retained a residual Cl^-^ transport activity.

### Repeatability of V_TE_ values

Repeatability between two series of measurements within the same group was good. We used Bland and Altman plots to define the limits of agreement in B6;129 WT and *cftr* ^-/-^ mice, making it possible to distinguish between simple variability and changes due to treatment. These data are essential for interpreting treatment effects, taking intra-animal variability into account.

We did not calculate coefficients of variance (%CV) for the V_TE_ data because the standard deviation did not vary with the mean and some means were close to zero, making interpretation unreliable [30], [31]. We did not determine correlation coefficients either, because these coefficients was not appropriate to this kind of analysis [22].

### Cutoff points for V_TE_ values

We were able to determine cutoffs for each of the V_TE_ parameters from ROC curves. We favored specificity over sensitivity. These cutoff points make it possible to classify V_TE_ measurements as belonging to a WT or CF profile, with a high degree of discrimination. This tool is crucial for preclinical studies of new drugs for cystic fibrosis treatment, particularly given the difficulties involved in interpreting the effects of treatment due to the potential residual activity of F508del-CFTR. The low-Cl^-^ cut-off, -2.15 mV, is the most relevant cutoff because it directly reflects correction of the CFTR defect. This is the first attempt, to our knowledge, to determine V_TE_ endpoints for preclinical studies.

### Applications for these cutoff points

We recently used the low-Cl^-^ cutoff to demonstrate the effect of keratin-8 siRNA treatment to restore F508del-CFTR activity [6]. It was hypothesized that keratin-8 interacts with F508del-CFTR and that disruption of this interaction would restore CFTR activity. Cl^-^ secretion exceeded the -2.15 mV cutoff for 50% of the treated mice and none of the control mice, establishing proof-of-concept for the treatment. F508del-CFTR rescue can further be demonstrated by inhibition of the response with a specific inhibitor, such as Inh-172.

In summary, we report here typical V_TE_ values for mice of two backgrounds not previously investigated: B6;129 and FVB. We show that our protocol for V_TE_ measurement is repeatable and we have determined V_TE_ cutoff values for distinguishing between CF and WT responses. This study constitutes an advance in the investigation of F508del-CFTR correctors/potentiators or ENaC hyperabsorption suppressors.

## Supporting Information

Table S1 **Forskolin response in WT and CF mice.** Values are voltage differences between 100 µM amiloride in low-Cl- solution perfusion and 100 µM amiloride plus 10 µM forskolin in low-Cl- solution perfusion (ΔV_TE_ _Forsk_).
(DOC)
