# Supplementary material for: Characterization of Nasal Potential Difference in cftr Knockout and F508del-CFTR Mice
Source: PLoS One. 2013 Mar 7;8(3):e57317. doi: 10.1371/journal.pone.0057317 (PMC3591431; doi:10.1371/journal.pone.0057317)
Supplement: Table S1 — Forskolin response in WT and CF mice. Values are voltage differences between 100 µM amiloride in low-Cl- solution perfusion and 100 µM amiloride plus 10 µM forskolin in low-Cl- solution perfusion (ΔVTE Forsk). (DOC) [file pone.0057317.s001.doc]

**Table S1 : Forskolin response in WT and CF mice.** Values are voltage differences between 100 µM amiloride in low-Cl^-^ solution perfusion and 100 µM amiloride plus 10 µM forskolin in low-Cl^-^ solution perfusion (ΔV_TE Forsk._).

| ΔV_TE Forsk_. (mV) | B6;129  WT  (*n* = 10) | FVB  WT  (*n* = 9) | FVB  F508del  (*n* = 8) |
| --- | --- | --- | --- |
|  |  |  |  |
| Mice 1 | 0.9 | 0.3 | 0.4 |
| Mice 2 | 0.3 | -2.7 | -1.9 |
| Mice 3 | -2.4 | -1.0 | 0.3 |
| Mice 4 | -2.0 | 0.3 | 0.5 |
| Mice 5 | 1.1 | 0.1 | 1.3 |
| Mice 6 | -0.7 | -0.3 | 0.8 |
| Mice 7 | 2.2 | 0.3 | -0.5 |
| Mice 8 | -3.0 | 0.3 | 0.8 |
| Mice 9 | 0.3 | 1.7 | - |
| Mice 10 | 2.8 | - | - |
|  |  |  |  |
|  |  |  |  |
| Median (IQR) | 0.3 (3.5) | 0.3 (1.0) | 0.45 (1.1) |
